# Novel miRNA Predicts Survival and Prognosis of Cholangiocarcinoma Based on RNA-seq Data and *In Vitro* Experiments

**DOI:** 10.1155/2020/5976127

**Published:** 2020-12-09

**Authors:** Yuan Yao, Dechao Jiao, Zaoqu Liu, Jianjian Chen, Xueliang Zhou, Zhaonan Li, Jing Li, Xinwei Han

**Affiliations:** ^1^Department of Interventional Radiology, The First Affiliated Hospital of Zhengzhou University, Zhengzhou, 450052 Henan, China; ^2^Academy of Medical Sciences, Zhengzhou University, Zhengzhou, 450052 Henan, China

## Abstract

Accumulating evidence has demonstrated that microRNAs (miRNAs or miRs) play an important role in the diagnosis and prognosis of tumors. In the case of cholangiocarcinoma (CCA), miRNAs may serve as potential tumor biomarkers and therapeutic targets. Based on The Cancer Genome Atlas (TCGA) database, fold change >2 was used to screen out miRNAs with differential expression in patients with CCA. Univariate and multivariate Cox regression analyses identified miR-3913-5p as an independent prognostic factor in patients with CCA. Overall survival and progression-free survival of patients with CCA were analyzed based on clinical data from TCGA database. In addition, four datasets were combined to identify 21 possible target genes of miR-3913, and Gene Ontology and Kyoto Encyclopedia of Genes and Genomes analyses were conducted to predict potential pathways and functions of the molecular target genes. Subsequently, the miRNAs associated with survival were selected to build the miRNA-mRNA expression network. Furthermore, the differential expression of miR-3913-5p in CCA cells and normal bile duct epithelial cells was confirmed through *in vitro* experiments. The possible target genes (RNF24 and SIGLEC) were further screened by reverse transcription-quantitative PCR. In addition, functional experiments showed that miR-3913-5p might be an oncogene that affects the proliferation and migration of CCA cells by inhibiting and mimicking miR-3913-5p. Therefore, miR-3913 may serve as a biomarker for the diagnosis and prognosis of patients with CCA.

## 1. Introduction

Cholangiocarcinoma (CCA) is the second most common malignancy of the hepatobiliary system, second only to hepatocellular carcinoma, and the incidence of CCA has been increasing in the past 40 years [1, 2]. Due to the anatomic location and growth pattern of CCA and the lack of clear diagnostic criteria for it, diagnosing this carcinoma is difficult [3]. In addition, this malignant tumor from the bile duct epithelium is highly aggressive, and patients have a poor survival prognosis [4]. Currently, CCA is diagnosed by combining clinical features, laboratory analysis, and imagological diagnosis [3, 5]. However, traditional diagnostic markers may not be particularly sensitive to tumor development. Some researchers have reported that the microRNA- (miR-) 15a/pPAI-2 axis may serve as a potential therapeutic target in patients with CCA [6]. Numerous studies have confirmed that lncRNAs may be therapeutic targets and biomarkers for CCA [7–9]. Moreover, exosomes derived from the bile may also serve as biomarkers for the early diagnosis of CCA due to their stable biological characteristics [10, 11]. Therefore, searching for effective and sensitive biomarkers is important to the early diagnosis and treatment of CCA.

MicroRNAs (miRNAs) are small single-stranded RNAs that measure ~22 nt and are processed from intracellular transcripts of hairpin structures. The processing and maturation of miRNAs undergo a spatial transition from the nucleus to the cytoplasm, and the whole process is coordinated by a variety of enzymes and helper proteins subjected to multilevel regulation and multistep precise reactions [12]. miRNAs regulate malignancies by binding to the partially complementary recognition sequences in the 3-untranslated region of mRNAs, and RISC is involved in the process. There are three main effects of miRNAs: transcriptional repression, mRNA cleavage, and mRNA degradation [13–17]. It has been reported that miRNAs can induce hOCT1 mRNA decay which, in turn, increases the sensitivity of CCA to sorafenib [18]. In addition, previous studies have confirmed that miR-494 is a regulator of the growth of CCA and is expected to become a new therapeutic target [19]. Lin et al. revealed the association between miRNAs and inflammation in CCA and may develop an miRNA-based treatment strategy via IL-6/STAT3 [20].

However, the role of miR-3913-5p in tumors has not been reported. The present study identified high expression of miR-3913 in CCA through The Cancer Genome Atlas (TCGA) database. The role of miR-3913 in the proliferation and migration of CCA has been demonstrated for the first time by bioinformatic analysis and *in vitro* experiments. The aim of this study was to develop CCA biomarkers to predict the survival and prognosis of patients with CCA based on miR-3913-5p.

## 2. Materials and Methods

### 2.1. TCGA Dataset of CCA

miRNA sequences and clinical information were downloaded from TCGA database (https://portal.gdc.cancer.gov/) according to the following inclusion criteria: (1) samples contained complete miRNA sequences and clinical information and (2) samples had prognostic information. Patients were excluded if they (1) lacked miRNA sequencing data, (2) did not have prognostic information, (3) were not primary cholangiocarcinoma, and (4) were missing clinical data. Until December 20, 2019, a total of 45 patients (36 patients with CCA and 9 benign controls) had been included.

### 2.2. Information Collection of the Validation Dataset

Two independent datasets downloaded from the Gene Expression Omnibus (GEO) database were used to verify the miRNA-3913-5p expression in patients with CCA (GSE85589 and GSE53992).

### 2.3. Principal Component Analysis (PCA) and Differential Gene Expression

PCA is a mathematical algorithm that can reduce data dimensions while retaining the vast majority of variables in a dataset [21]. The application of PCA makes the similarities and differences between samples from TCGA clearer and provides a reference for the classification of different samples. The 2,588 miRNAs associated with patients with CCA were analyzed with the Illumina HiSeq miRNA Seq platform (Illumina), and the R language package “edgeR” was used for calculation of differentially expressed miRNAs. ∣LogFC | >1 was defined as a difference in gene expression, and *P* < 0.05 was considered to indicate a statistically significant difference.

### 2.4. Selection of the Cut-Off Point and Survival Analysis

The R package “survminer” was utilized to determine the cut-off point for patient stratification into two groups. Overall survival (OS) and progression-free survival (PFS) were assessed in the high- and low-expression groups by Kaplan-Meier survival analysis (log-rank method). Those miRNAs with *P* < 0.05 were considered to exhibit a statistically significant difference in terms of survival and therefore were included in further analyses.

### 2.5. Construction of a Diagnostic and Prognostic Model as a Biomarker Based on miR-3913-5p

Tumor incidence from TCGA database was included as an outcome indicator, and the R package “pROC” was utilized to construct receiver operating characteristic (ROC) curves for evaluating the diagnostic model. Similarly, the survival status and survival time of the samples were extracted, and the R package “timeROC” was used to evaluate the accuracy of mir-3913-5p as a prognostic biomarker.

### 2.6. Target Gene Prediction and Further Analysis

The online tools miRDB (http://www.mirdb.org/), miRWalk (http://mirwalk.umm.uni-heidelberg.de/), TargetScan (http://www.targetscan.org/), and miRTarBase (http://mirtarbase.mbc.nctu.edu.tw/php/index.php) were used to predict the target genes of miRNA, and the intersection of these four online tools was selected by Venn diagram to further screen out more reliable target genes. Subsequently, Gene Ontology (GO) annotation and Kyoto Encyclopedia of Genes and Genomes (KEGG) pathway enrichment analyses were used to predict the functional enrichment and functional pathways of miRNA target genes. In addition, Gene-Set Enrichment Analysis (GSEA) was performed to further supplement the results of GO and KEGG analyses of miRNA target genes.

### 2.7. Cell Lines and Culture

HCCC-9810 and RBE from intrahepatic cholangiocarcinoma and HIBEpiC from normal bile duct epithelium were obtained from American Type Culture Collection. RPMI-1640 containing 10% fetal bovine serum (FBS) (Gibco; Thermo Fisher Scientific, Inc.) was used as complete medium for the above cells, which were cultured at 37°C in humid air under 5% CO_2_ conditions.

### 2.8. Cell Transfection

MiR-3913-5p mimics (micrON™ miRNA mimic, 50 nM), inhibitors (micrOFF™ miRNA inhibitor, 200 nM), negative control (NC), and inhibitor negative control (INC) were purchased from Guangzhou RiboBio Co., Ltd. Lipofectamine 3000 (Invitrogen; Thermo Fisher Scientific, Inc.) was used as a transfection carrier. The transfection efficiency was validated by reverse transcription-quantitative PCR (RT-qPCR) analysis.

### 2.9. RNA Isolation and RT-qPCR

Total RNA was extracted from the cell lines (HCCC-9810, RBE and HIBEpiC) using TRIzol reagent (Invitrogen; Thermo Fisher Scientific, Inc.). The extracted RNA was reverse transcribed using the PrimeScript™RT reagent Kit with gDNA Eraser (Takara Biotechnology Co., Ltd.), and the expression level of miR-3919-5p was measured with the SYBR Green PCR Master Mix kit (Beijing Solarbio Science & Technology Co., Ltd). U6 served as an internal reference to measure the relative miRNA expression by the 2^-*ΔΔ*Cq^ method. The primers for U6, miR-3913-5p, and target genes were designed and synthesized by Sunya. The following primer sequences were used: U6, forward (F) 5′-CTCGCTTCGGCAGCACA-3′ and reverse (R) 5′-AACGCTTCACGAATTTGCGT-3′; and miR-3913-5p, F 5′-CGCGCGTTTGGGACTGATCTTG-3′ and R 5′-AGTGCAGGGTCCGAGGTATT-3′. The sequences of the primers for the target genes are shown in Table S1.

### 2.10. Cell Counting Kit-8 (CCK-8) Assay

The CCA cell lines (HCCC-9810 and RBE) were transfected with miR-3913-5p mimics, miR-3913-5p inhibitors, NC, or INC and then seeded in 96-well plates with 3,000 cells per well. A total of 10 *μ*l CCK-8 solution (Abbkine Scientific Co., Ltd.) was added to each well and incubated for 2 h in a 5% CO_2_ incubator at 37°C. Finally, the absorbance at 450 nm was determined using the SpectraMax Absorbance Reader CMax Plus (Molecular Devices, LLC) at 0, 24, 48, 72, and 96 h. Each experiment was repeated ≥3 times.

### 2.11. Colony Forming Assay

The transfected cells were seeded in 6-well plates with 1,000 cells per well and then cultured in a 5% CO_2_ incubator at 37°C for 14 days. Cells were photographed, and the number of colonies with >50 *μ*m diameter was calculated. The experiment was repeated ≥3 times.

### 2.12. Transwell Assay for Migration

Twenty-four-well cell culture plates with 8 *μ*m inserts were purchased from EMD Millipore. For the migration assay, the HCCC and RBE cell lines (1 × 10^5^ cells per well) were added to the upper chamber in the presence of serum-free RPMI-1640 medium. A total of 500 *μ*l complete medium (RPMI − 1640 + 10%FBS) was added to the lower chamber. The 24-well plates were placed in a cell incubator and incubated at 37°C and 5% CO_2_ for 48 h. Next, cells in the upper compartment that failed to pass through the spoon were removed and washed with PBS 2-3 times. The cells that passed through the upper compartment and adhered to its lower surface were fixed with 4% tissue fixation solution (Elabscience Biotechnology Co., Ltd) for 0.5 h, stained with 0.1% crystal violet for 0.5 h, air dried at room temperature, and observed under the OLYMPUS BX43 (Olympus Corporation). Five fields of view were randomly selected for counting (magnification, ×100). The assay was carried out 3 times independently.

### 2.13. Statistical Analysis

The SPSS 20.0 software (IBM Corp.) was used for statistical analysis. Comparison between cell lines was done using one-way ANOVA test followed by post hoc test (Dunnett). The log-rank test was used to compare the survival of the high- and low-expression groups. Univariate/multivariate Cox multivariate hazard regression analyses were used to screen for survival-associated miRNAs and independent factors affecting prognosis based on clinical information. Two-way ANOVA test (Sidak's multiple comparisons test) was used to compare the cell proliferation. The Student's *t*-test was used to compare the differences between two groups. *P* < 0.05 was considered to indicate a statistically significant difference.

## 3. Results

### 3.1. Exploration and Verification of Gene Differential Expression

**Figure 1:**
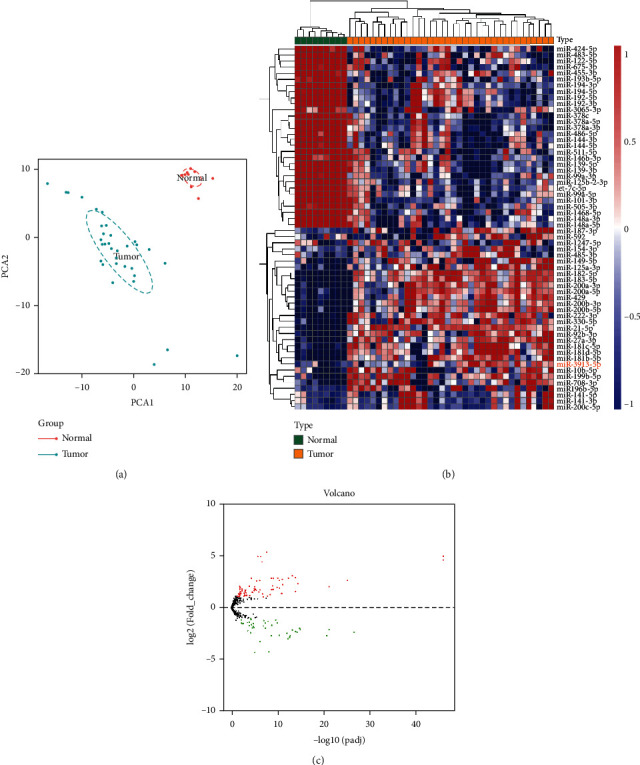
Differential expression of miRNAs in tumor tissues and controls: (a) principal component analysis (PCA) of samples; (b) miRNA clustering diagram with significant differences between samples; (c) volcano diagram of miRNA expression differences between samples.

**Figure 2:**
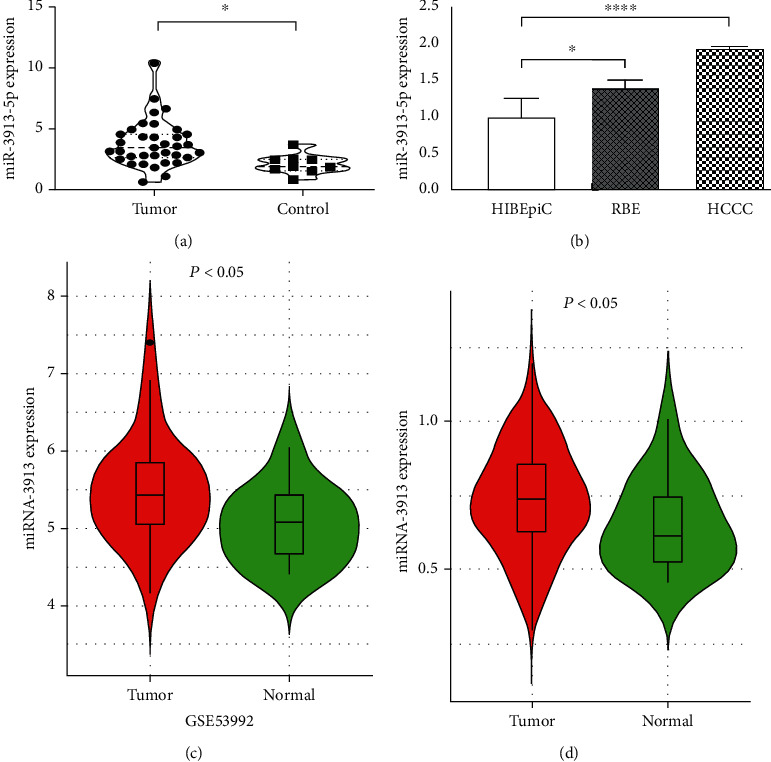
miR-3913-5p was upregulated in CCA tissues and cell lines: (a) analysis of the expression level of miR-3913-5p in CCA samples and normal tissues based on The Cancer Genome Atlas (TCGA) database; (b) analysis of the expression level of miR-3913-5p in cell lines (HIBEpiC, RBE, and HCCC); (c) analysis of the expression level of miR-3913-5p in CCA samples and normal tissues based on the GEO database (GSE53992); (d) analysis of the expression level of miR-3913-5p in CCA samples and normal tissues based on the GEO database (GSE85589). ^∗^*P* < 0.05, ^∗∗∗∗^*P* < 0.0001. CCA, cholangiocarcinoma; GEO, Gene Expression Omnibus.

Through PCA analysis, a PCA diagram was drawn, and similarities and differences were found between samples (Figure 1(a)). According to the PCA figure, we found that tumor samples were significantly different from the normal samples. This demonstrated the reliability of the data from TCGA and further verifies the robustness of the difference analysis results. In addition, 45 samples (36 cases of CCA and 9 controls) were selected by searching TCGA database (Table 1). Based on the sequencing results, miRNAs with ∣logFC | >1 were defined as CCA with differential expression in the control group. A total of 128 differentially expressed genes were obtained, 83 of which were upregulated and 45 of which were downregulated in CCA. The heat map of cluster analysis was used to visually reflect the differential expression of miRNAs in the two groups of samples: Red represents high expression, and green represents low expression (Figure 1(b)). Furthermore, a volcano diagram was drawn: Red dots represent high-expression molecules; green dots represent low-expression molecules, and black dots represent no significant difference in expression between cancer and normal tissues (Figure 1(c)). Through the exploration of TCGA database, miR-3913-5p was highly expressed in the tumor group (Figure 2(a)). RNAs from a normal bile duct epithelial and two bile duct cancerous cell lines were extracted, and the significantly high expression of miR-3913-5p in tumor cell lines was verified by RT-qPCR (Figure 2(b)). Finally, the high expression of this molecule was also confirmed in patients with CCA in two datasets from the GEO database (GSE53992 and GSE85589) (Figures 2(c) and 2(d)). Therefore, miR-3913-5p may be the oncogenic molecule of CCA.

### 3.2. Screening of miRNAs Associated with Survival

The miRNAs were correlated with the clinical data and included in univariate Cox regression analysis.  Table 2 shows the top 10 miRNAs, 3 of which were preliminarily considered to be correlated with survival time (*P* < 0.05). To further screen out miRNAs that independently affect survival, the above 3 miRNAs that may be correlated with survival were included in the multivariate Cox regression analysis, and the results showed that miR-3913-5p may be an independent factor influencing the survival time of patients with CCA (Table 3). In addition, the clinical data associated with OS were included in the univariate and multivariate analyses, which also confirmed that miR-3913-5p was an independent factor affecting patient survival (Table 4). It should be noted that, during the survival analysis, 3 outliers were artificially excluded. As the sample size was small, the survival analysis may be biased; therefore, more samples need to be included to corroborate the results.

### 3.3. Survival Analysis of miR-3913-5p and Potential Biomarkers for Diagnosis and Prognosis

**Figure 3:**
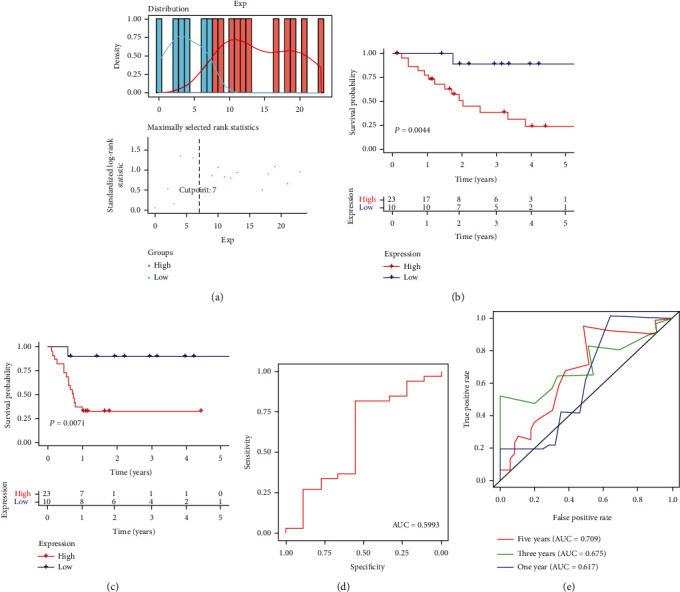
Kaplan-Meier survival curves and evaluation of predictive models. (a) Selection of the cut-off point by the R package “survminer.” (b) miR-3913-5p was associated with overall survival (OS) in patients with CCA. (c) miR-3913-5p was associated with progression-free survival (PFS) in patients with CCA. (d) ROC evaluated the accuracy of the diagnosis model. (e) ROC curve evaluated the accuracy of the prognostic model. ROC, receiver operating characteristic.

The optimal cut-off values were evaluated based on OS/progression-free survival (PFS) and miRNA expression data using the “survminer” package (Figure 3(a)). The results revealed that the OS in the high-expression group of miR-3913-5p was significantly lower than that in the low-expression group (*P* = 0.0044) (Figure 3(b)) and that the PFS in the high-expression group was lower than that in the low-expression group (*P* = 0.0071) (Figure 3(c)). In addition, based on the clinical information obtained from TCGA database, a ROC curve was drawn for evaluating diagnosis and prognostic model as a biomarker. The abscissa represents the false positive rate (specificity), and the ordinate represents the true positive rate (sensitivity). The area value under the ROC curve was between 0.5 and 1. The closer the AUC is to 1, the better the diagnostic effect is. The results showed that miR-3913-5p, as a biomarker for prognostic prediction, exhibited relatively high accuracy at 1 (AUC = 0.617), 3 (AUC = 0.675), and 5 years (AUC = 0.709) (Figure 3(e)). It should be noted that the accuracy of this model for diagnosis is not high (AUC = 0.5993) (Figure 3(d)). The accuracy of the diagnostic model may be affected by the insufficient sample size; therefore, more samples should be included in the future to confirm the value of miR-3913-5p as a biomarker.

### 3.4. Prediction and Validation of miR-3913-5p Target Genes

**Figure 4:**
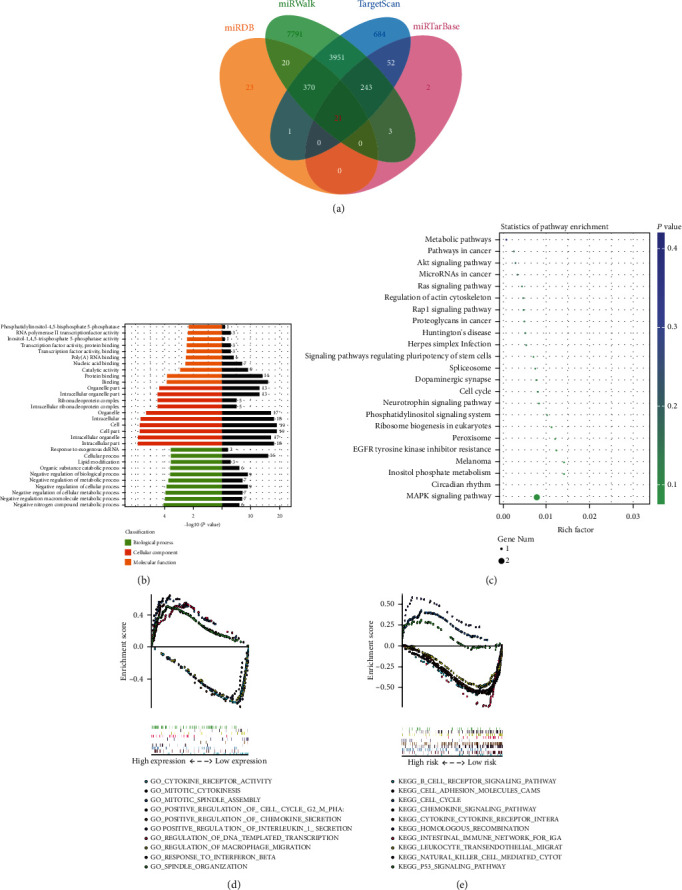
Prediction of miR-3913-5p target genes. (a) Target gene prediction of miR-3913-5p. The overlapping target genes of miRNA-3913-5p were predicted using the TargetScan, miRDB, miRWalk, and miRTarBase online analysis tools. (b) GO enrichment classification of miRNA target genes. (c) Bubble diagram of KEGG pathway classification of miRNA target genes. (d) Multi-GSEA analysis based on GO analysis and miR-3913-5p expression. (e) Multi-GSEA analysis based on KEGG analysis and miR-3913-5p expression. GO, Gene Ontology; KEGG, Kyoto Encyclopedia of Genes and Genomes; GSEA, Gene-Set Enrichment Analysis.

**Figure 5:**
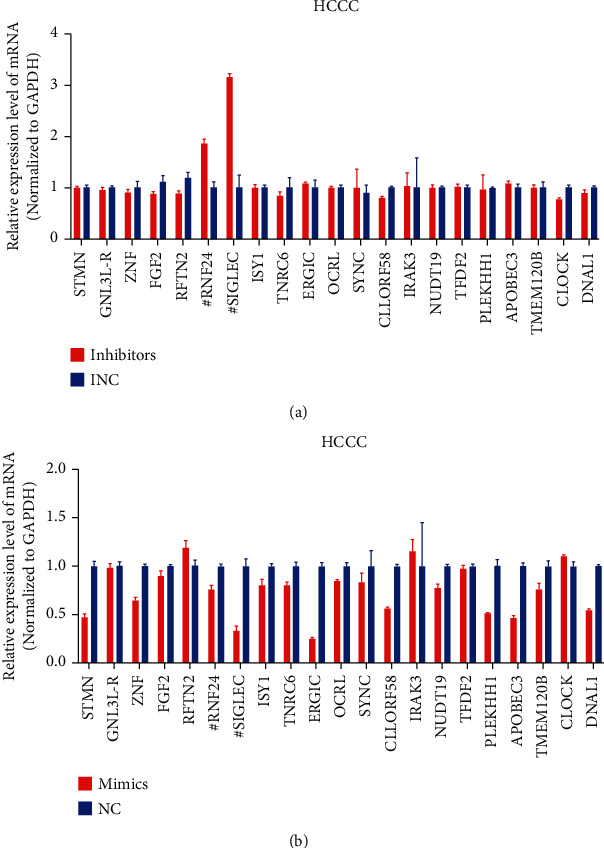
Validation of miR-3913-5p target genes by RT-qPCR: (a) expression of 21 overlapping target genes by downregulating miR-3913-5p-treated HCCC cells; (b) expression of 21 overlapping target genes by overexpressing miR-3913-5p-treated HCCC cells.

The intersection of the prediction results of target genes from the four databases (TargetScan, miRanda, clip-seq, and miRDB) revealed 21 reliable candidate target genes (Figure 4(a)). Subsequently, the overlapping target genes of miR-3913-5p were annotated and enriched through GO analysis. The results showed that target genes were mostly enriched in binding and protein binding for molecular functions. The enrichment range of target genes was relatively uniform and extensive in terms of cell composition, and the predicted function of target genes was mostly negative in regulating metabolism (Figure 4(b)). These analyses also explain the role of miR-3913-5p as an oncogenic gene in biological processes. To further predict the pathway, KEGG analysis was performed on consensus target genes (Figure 4(c)). The results showed that the pathway with the highest enrichment was the circadian rhythm, and the pathway with the highest number of target genes was the MAPK signaling pathway, which was closely associated with cell proliferation and metastasis [22, 23]. According to the expression of miR-3913-5p, the CCA samples were divided into high- and low-expression groups, and multi-GSEA analysis was conducted based on GO and KEGG, respectively (Figures 4(d) and 4(e)). Through analysis, it was found that the target genes of the high-expression group were associated with proliferation and metastasis function, while the low-expression group was associated with immune function, which could explain why the low-expression group mentioned above had a better prognosis than the high-expression group. To further screen out more reliable target genes, RNA was extracted from cell lines that inhibit and overexpress miR-3913-5p, and the expression of 21 candidate target genes was obtained by RT-qPCR. According to expression analysis, it was concluded that RNF24 and SIGLEC might be the target genes of miR-3913-5p (Figure 5). However, dual-luciferase reporter assays are needed to further determine this result.

### 3.5. Effect of miR-3913-5p on Proliferation and Migration In Vitro

**Figure 6:**
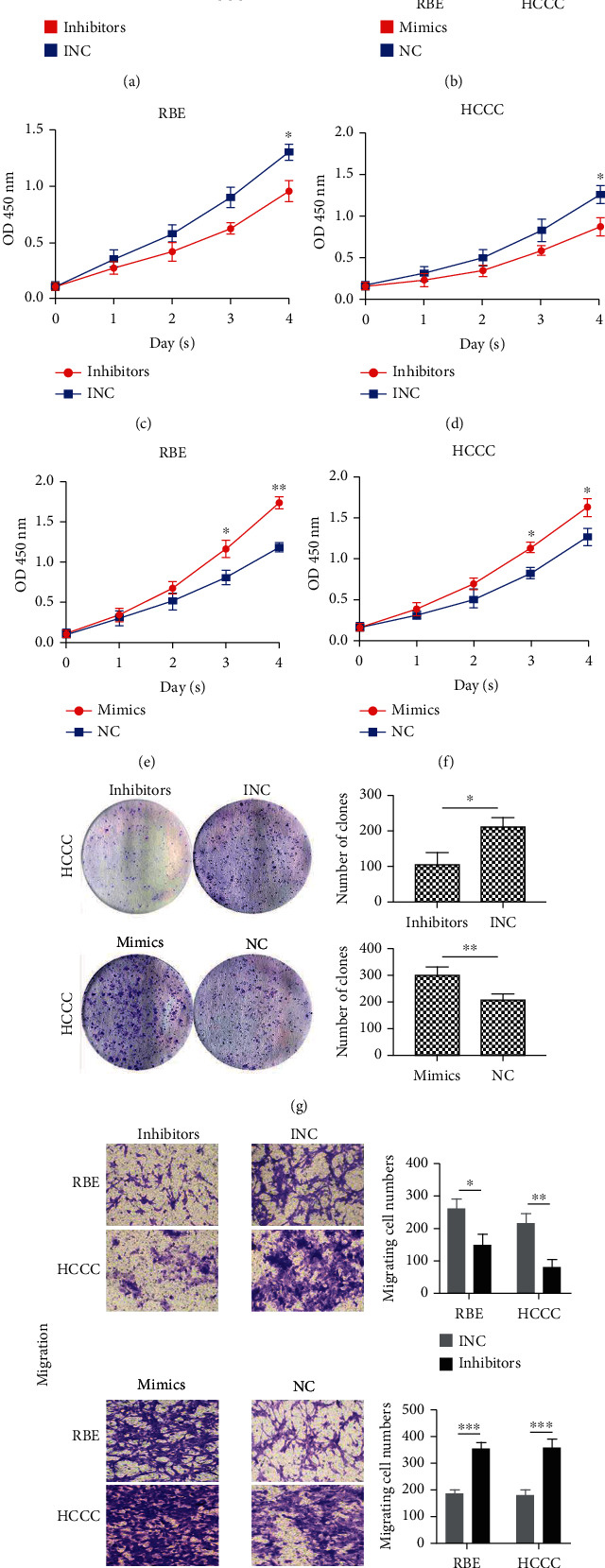
miR-3913-5p affects the proliferation and migration of CCA cells. (a, b) Verification of transfection efficiency by RT-qPCR. (c, d) Cell proliferation was determined in RBE and HCCC cells transfected with miR-3913-5p inhibitors or negative control. (e, f) Cell proliferation was determined in RBE and HCCC cells transfected with miR-3913-5p mimics or negative control. (g) Cell colony formation was affected by miR-3913-5p in HCCC cells. (h) miR-3913-5p affected the migration of the HCCC and RBE cell lines ^∗^*P* < 0.05, ^∗∗^*P* < 0.01, ^∗∗∗^*P* < 0.001, ^∗∗∗∗^*P* < 0.0001.

First, inhibitors of miR-3913-5p were transfected into HCCC and RBE cells, and successful transfection was confirmed by RT-qPCR (Figures 6(a) and 6(b)). Then, CCK-8 assays were conducted, and the results showed that this gene was inhibited and that the proliferation capacity of cells was significantly decreased (Figures 6(c) and 6(d)). Similarly, after mimic transfection was confirmed successfully, CCK-8 assays showed that the cell proliferation capacity was generally enhanced (Figures 6(e) and 6(f)). In addition, colony forming assays also revealed that cells that overexpressed miR-3913-5p had an enhanced colony forming capacity, while by inhibiting this gene, the colony forming ability decreased in HCCC cells (Figure 6(g)). Migration assays revealed that overexpression of miR-3913-5p promoted cell migration, whereas the inhibition of this gene reduced cell migration (Figure 6(h)). The results obtained for the two cell lines were consistent.

## 4. Discussion

Cholangiocarcinoma (CCA) includes a cluster of highly heterogeneous biliary malignant tumors that can arise at any point of the biliary tree. Their incidence is increasing globally, currently accounting for 15% of all primary liver cancers and 3% of gastrointestinal malignancies [24]. Dysregulation of miRNAs, along with functional or biomarker role, has been increasingly demonstrated in different tumor types. However, the role of miRNAs has not been fully elucidated in CCA. A recent study has been reported that have reported miRNA-137 suppresses the proliferation, migration, and invasion of cholangiocarcinoma cells by targeting WNT2B [25]. Similarly, miRNA-144 suppresses cholangiocarcinoma cell proliferation and invasion through targeting platelet activating factor acetylhydrolase isoform 1b [26]. A previous research has also showed that downregulated miRNA-876 in CCA tissue can inhibit tumor progression by targeting BCL-XL, which has potential therapeutic value for cholangiocarcinoma [27]. Therefore, miRNAs may be used as tumor suppressor genes to regulate the pathophysiological process of CAA. On the contrary, Zhang et al. have found that the expression of miR-30a-5p was increased in both CCA tissues and cells, and it could promote the proliferation of CCA cells by inhibiting SOCS3 [28]. The mechanisms of the above studies all focused on that miRNAs can inhibit, attenuate, or eliminate the expression of downstream genes. In recent years, accumulating studies have regarded miRNAs as the downstream of lncRNAs and circRNAs. They were used as sponges of miRNA, and the effect of miRNA on its target gene was downregulated [29–32].

The present study was the first to report the carcinogenic role of miR-3913-5p in tumors, especially in CCA. Samples of the CCA and control groups were extracted based on TCGA database and verified by the GEO database and *in vitro* experiments. A diagnostic and prognostic model of miR-3913-5p was constructed for the first time, which improved the diagnostic effectiveness of CCA to a certain extent, and it is beneficial to observe the prognosis of patients with CCA as a biomarker. However, due to the insufficient sample size, this novel biomarker may not be reliable and needs to be further confirmed by including a larger sample size or by using multiple miRNAs combined diagnostic models. Previous studies have confirmed that this diagnostic panel with multiple miRNAs can effectively improve the sensitivity of diagnosis, but an excessive number of molecules should not be included to avoid reducing the specificity of diagnosis [11, 33]. In addition, the miRNAs that were screened in the present study were closely associated with patient survival, and univariate and multivariate Cox regression analyses were used to identify miRNAs that independently affected patient survival. Our study combined the clinical information of the samples with the screened miR-3913-5p for univariate and multivariate analyses, further identifying this molecule as an independent factor affecting patient survival. Then, the survival curve of OS and FPS in patients with CCA was plotted, which suggested the use of this molecule as a prognostic indicator.

To further explore the molecular mechanism of miR-3913-5p, the present study focused on the prediction of target genes of miR-3913-5p and further explored the function of target genes through GO, KEGG, and GSEA analyses. Since the majority of the target genes were associated with negative regulation of cell metabolism, it was shown that miR-3913-5p might be a potential tumor promotor. Furthermore, according to the two candidate target genes SIGLEC10 and RNF24, it was predicted that SIGLEC10 might affect the metastasis of CCA by changing the cell adhesion ability through the above analysis. Some studies have confirmed that the combination of vascular adhesion protein-1 and SIGLEC10 could affect cell adhesion [34]. RNF24 may affect the proliferation of tumor cells by affecting cell metabolism. Some researchers have reported that RNF24 plays a role in the progression of Barrett's esophagus to esophageal adenocarcinoma [35]. To verify these predictions, CCK-8, Transwell, and colony forming assays were conducted, and the results showed that overexpression of miR-3913-5p was associated with increased proliferation, clonal formation, and migration of CCA cells. By suppressing the gene, the results were reversed. Therefore, the poor prognosis of patients in the high-expression group might be associated with the proliferation and metastasis of tumor cells mediated by miR-3913-5p. This molecule may also be used as a therapeutic target to effectively prolong the survival of patients.

However, the present study has several limitations. First, the sample size of TCGA was too small, the diagnostic panel was relatively unstable, and the prediction might be biased. Second, the sequencing results were obtained from the CCA tissues and the corresponding control group which may not accurately reflect the levels of miRNAs in saliva, serum, urine, or stool. Hence, we need to further explore the expression of miR-3913-5p in the above samples to facilitate the detection of its expression level. In addition, the experimental validation only included *in vitro* experiments. Due to the complexity of the body, *in vivo* experiments are needed to further confirm the role of miR-3913-5p in the progression of CCA.

## 5. Conclusion

In conclusion, we have demonstrated that miR-3913-5p is upregulated in CCA cell lines, and high expression of this gene is associated with poor prognosis based on TCGA database. Especially, it has been served as an independent factor of the prognosis of CCA according to the univariate and multivariate analyses. Furthermore, we have revealed for the first time the role of this oncogene in promoting the proliferation and migration of CCA cells, and its potential target genes are SIGLEC10 and RNF24. Taken together, these findings describe a novel and promising biomarker for prognosis in CCA.

## Figures and Tables

**Table 1:** Clinical characteristics of the cholangiocarcinoma patients in TCGA database.

Characteristics	Number of cases (%)
Age (y)	
≤60	14 (38.89)
>60	22 (61.11)
Gender	
Male	16 (44.44)
Female	20 (55.56)
Clinical stage	
I + II	28 (77.78)
III + IV	8 (22.22)
T classification	
T1 + T2	31 (86.11)
T3 + T4	5 (13.89)
N classification	
N0	26 (72.22)
N1	5 (13.89)
NX	5 (13.89)
Metastasis	
No	28 (77.78)
Yes	5 (13.89)
MX	3 (8.33)
Histological type	
Intrahepatic	30 (83.33)
Hilar + distal	6 (16.67)
Total	36 (100)

**Table 2:** Univariate Cox analysis list.

ID	Coef	Exp (coef)	Se (coef)	*z*	*P* value
hsa-miR-22-3p|MIMAT0000077	1.07*E*-05	1.000011	4.70*E*-06	2.272639	0.023048
hsa-miR-3913-5p|MIMAT0018187	0.054562557	1.056079	0.024014	2.272158	0.023077
hsa-miR-3065-3p|MIMAT0015378	0.003287932	1.003293	0.001497	2.196994	0.028021
hsa-miR-10b-5p|MIMAT0000254	-0.000131775	0.999868	6.88*E*-05	-1.91432	0.05558
hsa-miR-224-5p|MIMAT0000281	0.007645179	1.007674	0.004	1.911261	0.055971
hsa-miR-30a-5p|MIMAT0000087	-2.34*E*-05	0.999977	1.27*E*-05	-1.83405	0.066647
hsa-miR-301a-3p|MIMAT0000688	-0.195737289	0.822228	0.119029	-1.64445	0.100083
hsa-miR-92b-3p|MIMAT0003218	0.007430323	1.007458	0.004712	1.576799	0.114842
hsa-miR-181a-5p|MIMAT0000256	0.000163027	1.000163	0.000105	1.553821	0.120227
hsa-miR-152-3p|MIMAT0000438	-0.006971629	0.993053	0.004494	-1.55117	0.120862

**Table 3:** Multivariate Cox analysis list.

ID	Coef	Exp (coef)	Se (coef)	*z*	Pr (>|*z*|)	Lower 0.95	Upper 0.95
′hsa-miR-22-3p|MIMAT0000077′	1.346994	3.845847	0.625932	2.151982	0.031399	1.127711	13.11554
′hsa-miR-3065-3p|MIMAT0015378′	0.172876	1.188719	0.167997	1.029042	0.30346	0.855224	1.65226
′hsa-miR-3913-5p|MIMAT0018187′	0.955322	2.599508	0.326191	2.928723	0.003404	1.37163	4.926576

**Table 4:** Univariate and multivariate analyses of parameters associated with overall survival.

Variables	Univariate analysis		Multivariate analysis	
	HR (95% CI)	*P* value	HR (95% CI)	*P* value
miR-3913-5P	1.092 (1.041-1.146)	<0.001	1.154 (1.038-1.284)	0.008
Age	1.019 (0.970-1.070)	0.460		
Gender	1.604 (0.513-5.015)	0.416		
Histologic grade	1.663 (0.432-6.397)	0.459		
T	1.178 (0.569-2.435)	0.659		
N	2.572 (0.659-10.038)	0.174		
M	1.372 (0.249-6.396)	0.688		
Platelet	2.463 (0.691-8.785)	0.165		
Vascular invasion	1.963 (0.414-9.317)	0.396		
Stage	1.298 (0.824-2.046)	0.260		

HR, hazard ratio; CI, confidence interval.

## Data Availability

The data used to support the findings of this study are available from the corresponding author upon request.

## Supplementary Materials

Supplementary Materials Table S1: primers of target genes for qRT-PCR.

## References

[1] Welzel T. M., McGlynn K. A., Hsing A. W., O'Brien T. R., Pfeiffer R. M. (2006). Impact of classification of hilar cholangiocarcinomas (Klatskin tumors) on the incidence of intra- and extrahepatic cholangiocarcinoma in the United States. *JNCI: Journal of the National Cancer Institute*.

[2] Saha S. K., Zhu A. X., Fuchs C. S., Brooks G. A. (2016). Forty-year trends in cholangiocarcinoma incidence in the U.S.: intrahepatic disease on the rise. *The Oncologist*.

[3] Blechacz B., Komuta M., Roskams T., Gores G. J. (2011). Clinical diagnosis and staging of cholangiocarcinoma. *Nature Reviews. Gastroenterology & Hepatology*.

[4] Clements O., Eliahoo J., Kim J. U., Taylor-Robinson S. D., Khan S. A. (2020). Risk factors for intrahepatic and extrahepatic cholangiocarcinoma: a systematic review and meta-analysis. *Journal of Hepatology*.

[5] Rizvi S., Khan S. A., Hallemeier C. L., Kelley R. K., Gores G. J. (2018). Cholangiocarcinoma - evolving concepts and therapeutic strategies. *Nature Reviews. Clinical Oncology*.

[6] Utaijaratrasmi P., Vaeteewoottacharn K., Tsunematsu T. (2018). The microRNA-15a-PAI-2 axis in cholangiocarcinoma-associated fibroblasts promotes migration of cancer cells. *Molecular Cancer*.

[7] Zhang C., Li J.-Y., Tian F.-Z. (2018). Long noncoding RNA NEAT1 promotes growth and metastasis of cholangiocarcinoma cells. *Oncology Research Featuring Preclinical and Clinical Cancer Therapeutics*.

[8] Xu Y., Yao Y., Jiang X. (2018). SP1-induced upregulation of lncRNA SPRY4-IT1 exerts oncogenic properties by scaffolding EZH2/LSD1/DNMT1 and sponging miR-101-3p in cholangiocarcinoma. *Journal of Experimental & Clinical Cancer Research*.

[9] Wang W.-T., Ye H., Wei P.-P. (2016). LncRNAs H19 and HULC, activated by oxidative stress, promote cell migration and invasion in cholangiocarcinoma through a ceRNA manner. *Journal of Hematology & Oncology*.

[10] Severino V., Dumonceau J. M., Delhaye M. (2017). Extracellular vesicles in bile as markers of malignant biliary stenoses. *Gastroenterology*.

[11] Li L., Masica D., Ishida M. (2014). Human bile contains microRNA-laden extracellular vesicles that can be used for cholangiocarcinoma diagnosis. *Hepatology*.

[12] Kim V. N. (2005). MicroRNA biogenesis: coordinated cropping and dicing. *Nature Reviews Molecular Cell Biology*.

[13] Fabian M. R., Sonenberg N. (2012). The mechanics of miRNA-mediated gene silencing: a look under the hood of miRISC. *Nature Structural & Molecular Biology*.

[14] Fabian M. R., Cieplak M. K., Frank F. (2011). miRNA-mediated deadenylation is orchestrated by GW182 through two conserved motifs that interact with CCR4–NOT. *Nature Structural & Molecular Biology*.

[15] Lim L. P., Lau N. C., Garrett-Engele P. (2005). Microarray analysis shows that some microRNAs downregulate large numbers of target mRNAs. *Nature*.

[16] Meister G., Landthaler M., Patkaniowska A., Dorsett Y., Teng G., Tuschl T. (2004). Human Argonaute2 mediates RNA cleavage targeted by miRNAs and siRNAs. *Molecular Cell*.

[17] Yekta S. (2004). MicroRNA-directed cleavage of HOXB8 mRNA. *Science*.

[18] Lozano E., Macias R. I. R., Monte M. J. (2019). Causes of hOCT1-dependent cholangiocarcinoma resistance to sorafenib and sensitization by tumor-selective gene therapy. *Hepatology*.

[19] Olaru A. V., Ghiaur G., Yamanaka S. (2011). MicroRNA down-regulated in human cholangiocarcinoma control cell cycle through multiple targets involved in the G1/S checkpoint. *Hepatology*.

[20] Lin K.-Y., Ye H., Han B.-W. (2016). Genome-wide screen identified let-7c/miR-99a/miR-125b regulating tumor progression and stem-like properties in cholangiocarcinoma. *Oncogene*.

[21] Jolliffe I. T., Cadima J. (2016). Principal component analysis: a review and recent developments. *Philosophical Transactions of the Royal Society A: Mathematical, Physical and Engineering Sciences*.

[22] Peng W. X., Huang J. G., Yang L., Gong A. H., Mo Y. Y. (2017). Linc-RoR promotes MAPK/ERK signaling and confers estrogen-independent growth of breast cancer. *Molecular Cancer*.

[23] Han B., Zhao J. Y., Wang W. T., Li Z. W., He A. P., Song X. Y. (2017). Cdc42 promotes Schwann cell proliferation and migration through Wnt/*β*-Catenin and p38 MAPK signaling pathway after sciatic nerve injury. *Neurochemical Research*.

[24] Banales J. M., Marin J. J. G., Lamarca A. (2020). Cholangiocarcinoma 2020: the next horizon in mechanisms and management. *Nature Reviews Gastroenterology & Hepatology*.

[25] Chen T., Lei S., Zeng Z. (2020). MicroRNA-137 suppresses the proliferation, migration and invasion of cholangiocarcinoma cells by targeting WNT2B. *International Journal of Molecular Medicine*.

[26] Yang R., Chen Y., Tang C. (2014). MicroRNA-144 suppresses cholangiocarcinoma cell proliferation and invasion through targeting platelet activating factor acetylhydrolase isoform 1b. *BMC Cancer*.

[27] Ursu S., Majid S., Garger C. (2019). Novel tumor suppressor role of miRNA-876 in cholangiocarcinoma. *ONCOGENESIS*.

[28] Zhang J. W., Wang X., Li G. C. (2020). MiR-30a-5p promotes cholangiocarcinoma cell proliferation through targeting SOCS3. *Journal of Cancer*.

[29] Li D., Tang Z., Gao Z., Shen P., Liu Z., Dang X. (2020). Circular RNA CDR1as exerts oncogenic properties partially through regulating MicroRNA 641 in cholangiocarcinoma. *Molecular and Cellular Biology*.

[30] Xu Y., Yao Y., Liu Y. (2019). Elevation of circular RNA circ_0005230 facilitates cell growth and metastasis via sponging miR-1238 and miR-1299 in cholangiocarcinoma. *Aging (Albany NY)*.

[31] Guan C., Zhao Y., Wang W. (2020). Knockdown of lncRNA SNHG20 suppressed the proliferation of cholangiocarcinoma by sponging miR-520f-3p. *Cancer Biotherapy and Radiopharmaceuticals*.

[32] Peng L., Liu Y. H., Nie S., Gao M. (2020). LncRNA CASC2 inhibits cell proliferation, metastasis and EMT through miR-18a/SOCS5 axis in cholangiocarcinoma. *European Review for Medical and Pharmacological Sciences*.

[33] Cao J., Sun L., Li J. (2018). A novel three-miRNA signature predicts survival in cholangiocarcinoma based on RNA-Seq data. *Oncology Reports*.

[34] Kivi E., Elima K., Aalto K. (2009). Human Siglec-10 can bind to vascular adhesion protein-1 and serves as its substrate. *Blood*.

[35] Wang X. W., Wei W., Wang W. Q., Zhao X. Y., Guo H., Fang D. C. (2014). RING finger proteins are involved in the progression of Barrett esophagus to esophageal adenocarcinoma: a preliminary study. *GUT LIVER*.

