# Supplementary material for: Novel miRNA Predicts Survival and Prognosis of Cholangiocarcinoma Based on RNA-seq Data and In Vitro Experiments
Source: Biomed Res Int. 2020 Dec 9;2020:5976127. doi: 10.1155/2020/5976127 (PMC7787740; doi:10.1155/2020/5976127)
Supplement: Supplementary Materials — Table S1: primers of target genes for qRT-PCR. [file 5976127.f1.docx]

Supplementary table I Primers of target genes for qRT-PCR

| Genes | Primers |
| --- | --- |
| ERGIC1 | F: CAGACTCACCTCCAACCCCCT |
|  | R: GCCACTCTTGTCCTCATAAACCG |
| SIGLEC10 | F: GGGTGGAGAGAGGAAGCTATGTGA |
|  | R: CAGGTGAGGTCGGTGTTGTGG |
| TNRC6A | F: AATGGTGCTGGGCTGTCGGG |
|  | R: GCTAATTCCTCGGGGGTCGC |
| ISY1-F | F: AGAGAGAGAGGCTCGGCTGG |
|  | R: CTCGGTGACTGCATAGATGTTGAT |
| STMN1 | F: GGTGAAAGAACTGGAGAAGCGTG |
|  | R: TTGGAGGGGAAAGGGGGAAT |
| GNL3L | F: CCAGGTGAAGGTTCCAAGGG |
|  | R: GGGGTGAACAAAATGAGGTGC |
| ZNF280C | F: CTGTTACTGTTGAGAATGCGTC |
|  | R: TGCTGGTATGTCTTGGGATG |
| FGF2 | F: AAGAGCGACCCTCACATCAAG |
|  | R: CCGTAACACATTTAGAAGCCAGT |
| RFTN2 | F: TTCAACCTCCTGGGCCTATC |
|  | R: CAGCCAATCAGCATCAAGTGTA |
| RNF24 | F: GAGATGAGTTGGGGATTTGC |
|  | R: ACAGGGGACACACTTTACGA |
| OCRL | F: GACTCTGCTTATGATCCCCG |
|  | R: TTGGCATTGACGCTATTGTAT |
| SYNC | F: CAAGGGCGGAGAAATCTAACC |
|  | R: TGGACACACTGCTGGAAACG |
| C11orf58 | F: GAGGCAGCAGACTTGGGTAA |
|  | R: TTCGGAAGTGAGATGTTGATTTGT |
| IRAK3 | F: GCCTGGGAGCAGTGTTGAGT |
|  | R: GACATTGGCTGTTTCCTTGA |
| NUDT19 | F: CCACCCCCACAGTTCTACG |
|  | R: TGGACCATCCCATCAGCA |
| TFDP2 | F: AGATAGAGAAGCAGAGGCGG |
|  | R: TACCAGGTTTTTGAAAGCGA |
| PLEKHH1 | F: TGCGTAACCCCTTCCACCAC |
|  | R: AGAGCCGTCAAAACCAACCA |
| APOBEC3F | F: CTTCAGAAACACAGTGGAGCGA |
|  | R: CCAGACGGTATTCCGACGAG |
| TMEM120B | F: TGTTCGTACTGGCGTTCACC |
|  | R: GCTGCTTTGTCTTGCCTCTGT |
| DNAL1 | F: CCTGAATGGCTTAAAAAACT |
|  | F: TCTTCTAATGTGTCCCCTACTG |
| CLOCK | F: ATGTCCCAGTTTCAGTTTTCA |
|  | R: AGTTCTTCTTGTTGCCGATG |
